# Meteorological Observations

**Published:** 1859-04

**Authors:** J. G. Westmoreland

**Affiliations:** Smithsonian Institution, March, 1859, at Atlanta, Ga.


					﻿§ ll 2
&	*	“	*	72 ZZ;Z
1________________________________________
•' »J.....'
•|A	j I	jg
’■e	,	1^*
«<■<=>	a
S rd	(	      =
~	,! --------------------------------------------------O
g> o i!	•	•	GJ
R	-<	K H' > & jxj w' > > • k • fe’ M &: £ . pt • w . a . • . a . . £t ' a
© Jg	,	02	02^^	aj’*Hj’*a2^^	•	• • tZ2	.02 ,<Z2^^0	.02 02 a
C0-<	t-	™	02^^^’*^	02	^	02	02	_,
'S
•~ ©	I
R R> I ------;----------------------- I
&2 <s ;	,>a
S'.	fl
0 ' S I	°
" >	pi ,02®0®50®010‘<:io0ioo,o©>oo>o©>ooooqoqoo© S
©	rH	_	-
2s?	S
pj H S .	'	~------------------------1
®	“	S	2
°	3	*	10	13 c ° S c 13 °1010 ° 2 2 ° “"" 215 ® ’■’ °1-'3 c ° ° ®	>« o o c	5
u o’	1-1	-2
I	v
R S	I ___________________________________________________________________________________     ®
„g .•§	   .-3
£ $1	1	g	a
2 LS	<	°“®20022l®0l0v:,0102002c>0‘®‘®0000'®”®^®‘®^*
•£S	t,
CO	33
g J I-----y-------------------------------------------------,s>
§	££	-s	S	S8	2	8	8	®
____«____00	Q’P	©	w ©	O	o	©
*>I I s'	S	S^SSS^SSSSSSSSSSS^^gSSSSSSSS^SS
Ji	H	©
g5 g “5-----------------~-----------------------------------s
■g . 2 S^SSSSSSaSggSSgSSSSgSSSHSSgSggg 2
~ « ■, cd —---------------------- --- 5
s <5 g	~ ~ ~ s
•|.g h x 2=^^2^S®^^S^gS3®^gw®gg§3SM®S©£® 5
g § I ----Sr------------------------------------------------M
S	S S 13 S S P m X?	J? 2 ° P 0 2 *00»CC010000 O CO
Co —«	r-i7MHHa:ajcrCOQ . co O O Ci . r-< l^ O Q e. O CO N X N O t—< r-<
<y»	Ch	'-— Ci Ci Ci Ci Ci Ci Ci 'O Ci O’ O Ci O' <O Ci Ci Ci O' Ci’ Ci Ci Ci Ci Ci Ci O> ci ers 0*
O	g	COCQOMCq^OlQqCMaqcO^CQCQClCQCQGqSScOciSSSSSSSw x
i	——-----------------------—---— ...................
*2	!	a	s	« 2 12 2 2 So S li 5 53 2 S 10 22 °0 £J co >® '© 00 iccoocoiaoio
?	ta—I	□ r-< H H 71 CO LO Is* Q 0 Q O r-< r—' X Q O CO XHQCOCCCOC
'	Cj1	Q.	Ci Ci Ci Ci Ci Ci O Ci O’ O’ Ci Ci <O Ci Ci Ci c**^ (^» c*j .*) g* z*> fT\ z-r, z-^-	1
.	o	WW^^^^^^^W^COCO^COCOCIOKNWCQCqcO.lNOlO^ClW^IOl
»2 I H------;--------------------—-—---—--------------------
S’	JS	X	S2i2o£252 “5«’ © 1© © 10 io ci 10 to © uo
R	PQ	.	, 01 co i'-© c© © © .(©Or-< o © <© eroo © © <© i- t— cc o —r © (©
R	■*	2 2 S S ® S ?; £ 2 £; ® 2' ® ® ® ® ® ® ® t © -i © © © © g c; © ©
-m	M K c-rci .4 u jq N co ;K4 cc ci ci co m ?; ci co a ci ci n c-i c; ci m n ci
Day of Mo. I rt w	>■• ® © ® — ci co «o e hx © c — ci co -f >6 © i - oo © a
1	"	‘	'"’r-l rr m ri rrn rr r-l m M Cl Cl Cl Cl Cl Cl Cl Cl Cl OO CO
' J Ahl.’. ’	. t
				

